# The Saliva as a Test for the Functional Disorders of the Liver

**Published:** 1888-06

**Authors:** 


*The Saliva as a test for the Functional Disorders of the Liver.* By SAMUEL FENWICK, M.D., F.R.C.P. Pp. 78. London: J. & A. CHURCHILL. 1887.

Dr. Fenwick here presents us with an elaborate physiological study of an available secretion as a means of clinically testing the manner in which an important gland, whose secretions are not available, discharges its functions. The study relates to the extremely important question of how far change in structure necessitates change of function—one which we believe to be at the root of all future progress in medicine. As guides to treatment, the very first changes in function produced by diseases as studied by the physiologist in the laboratory must supplement, if they do not supersede, the terminal ravages in the destroyed organ as placed before the pathologist.

Using the amount of sulphocyanide in the saliva as his guiding index to the activity of the liver in nutrition, Dr. Fenwick has worked out with supreme patience and great thoroughness several very important conclusions. The results achieved in this small department of research are well worthy of study; the work is, perhaps, even more valuable as indicating a comparatively new and highly important line of investigation.

